# Morning Pseudoneutropenia in a Patient With Borderline Personality Disorder Treated With Clozapine

**DOI:** 10.1192/bjo.2022.99

**Published:** 2022-06-20

**Authors:** Tongeji Tungaraza

**Affiliations:** Priory Healthcare, Birmingham, United Kingdom

## Abstract

**Aims:**

Neutropenia associated with clozapine affects up to 3% of patients. For the purpose of clozapine treatment, absolute neutrophil count (ANC) between 1.5–2.0 × 109/L is considered as amber result; requiring twice-weekly blood sampling until when it returns to normal. Interestingly, some patients on clozapine may develop transient neutropenia also known as Morning Pseudoneutropenia (MPN), or pseudoneutropenia. It is a phenomenon where normal diurnal variation of circulating white blood cells (WBC) and in particular ANC become more accentuated. In these patients, blood samples taken in the morning would tend to have amber results, while blood samples taken on the same day in the afternoon will have normal ANC. A case is reported where a patient with severe emotionally unstable personality disorder (EUPD) developed MPN 38 days after clozapine initiation.

**Methods:**

AA is a 19-year-old white lady with a diagnosis of severe EUPD. Prior to starting clozapine, AA had tried several oral and depot antipsychotics, antidepressants and lithium without success. AA was started on clozapine. Her initial pre-clozapine blood count taken in the morning was WBC-5.8 x109/L and her ANC was 2.7 x109/L. AA improved quickly on clozapine. However, five weeks later, her first amber report was received. AA went on to have another six amber results before MPN was suspected. AA blood sampling was moved to the afternoon. There were no more amber results thereafter.

**Results:**

To my knowledge, this is the first published case of a patient with EUPD treated with clozapine who went on to develop MPN. Recurrent amber results with samples taken before mid-day should alert clinicians of this phenomenon. Of course, other causes of neutropenia need to be excluded.

It is noteworthy that most reported cases in literature though few, involves patients with treatment resistant schizophrenia. Being aware of this phenomenon may raise our suspicion index and hopefully reduce the need for frequent repeated blood tests and the need to stop clozapine given the risk of relapse and its implications.

Few cases have also been reported in patients with physical health problems such as Grave's disease, systemic lupus erythematosus, and lymphangioleiomyomatosis. Medications that have been implicated to potentiate ANC diurnal variation including clozapine, risperidone, prednisolone, methimazole, and sirolimus.

**Conclusion:**

Morning pseudoneutropenia is an accentuated normal diurnal variation of WBC. Early detection by collecting samples in the afternoon when there are recurring amber results can reduce unnecessary repeated blood tests and can prevent premature discontinuation of clozapine.

